# Environmental Recovery of Nosocomial Bacteria in a Companion Animal Shelter Before and After Infection Control Procedures

**DOI:** 10.3389/fvets.2020.608901

**Published:** 2021-01-20

**Authors:** Sara Horsman, Hester Rynhoud, Xiaoyan Zhou, Ricardo J. Soares Magalhães, Justine S. Gibson, Erika Meler

**Affiliations:** ^1^School of Veterinary Science, The University of Queensland, Gatton, QLD, Australia; ^2^Children's Health and Environment Program, Child Health Research Centre, The University of Queensland, Brisbane, QLD, Australia

**Keywords:** nosocomial bacteria, animal shelter, companion animals, environmental bacterial contamination, animal movements, cleaning and disinfection practices

## Abstract

While the effects of cleaning and disinfection practices on the reduction of environmental nosocomial bacteria are well-established in human and large animal veterinary hospitals, how animal movements within animal health care facilities influence environmental bacterial recovery is poorly understood. During three consecutive weeks, 155 electrostatic wipes were collected from the environment pre- and post-cleaning only or following disinfection from seven target locations within an animal shelter. All samples were cultured, and isolates were identified using a matrix-assisted laser desorption ionization—time of flight mass spectrometry. Social network analysis of animal movements during the sampling period was performed to estimate the level of connectivity of the seven target locations. The relationship between bacterial levels and connectivity estimates of the target locations were investigated using a negative binomial regression model with a random effect of sampling areas. Overall, our results indicate a significant reduction in the total bacterial contamination with disinfection when compared to cleaning only [Coefficient (Coef.) = −1.72, 95% Confidence Interval (CI) = −3.09, −0.34, *P* = 0.015]. Higher total bacterial contamination was significantly more likely in sampled areas with less animal movement connectivity (Coef. = −0.32, 95% CI = −0.49, −0.15, *P* ≤ 0.001). *Pseudomonas aeruginosa* and ampicillin resistant *Enterobacteriaceae* (*Escherichia coli, Enterobacter* spp. and *Klebsiella* spp.) were present in the animal holdings and in the shelters' veterinary clinic environment at all sampling times. Our findings demonstrate that cleaning followed by disinfection practices are effective at reducing environmental bacterial levels. Areas with less animal connectivity are more likely to have a higher bacterial contamination. These areas could represent environmental reservoirs for bacterial infection and should be targeted with effective cleaning and disinfection protocols.

## Introduction

Nosocomial infections are a growing concern in both human and veterinary medicine. Commonly isolated bacteria causing nosocomial infections in dogs and cats include: methicillin resistant *Staphylococcus aureus* (MRSA), methicillin resistant *S*. *pseudintermedius* (MRSP), *Pseudomonas aeruginosa* and multidrug resistant (MDR) *Enterobacteriaceae* (1–4). These potential pathogens present in the environment of veterinary clinics may be transmitted to animals via direct contact, veterinary personnel, other animals, and may cause infections such as surgical site, urinary tract, and bloodstream infections (5).

Exposure to environmental bacterial contamination can contribute to the carriage of bacteria in dogs and cats (5). As such, studies have investigated the presence of environmental bacteria in veterinary clinics but often only at one or two sampling times (6–8). Potential nosocomial bacterial pathogens have been isolated from surfaces that animals and humans frequently come in contact with in veterinary clinics, including: animal cages, water bowls, floors, water taps, door handles, treatment, and surgical benches (1, 6–9). Studies sampling small animal veterinary clinic environments have reported the bacterial recovery of *Escherichia coli* ranging from 76 to 89.5% (1, 10), MRSA from 1.4–16% (7, 9), MRSP from 2 to 13.8% (6, 11) and *P*. *aeruginosa* isolated from 30% (3/10) of the sampled environments (4). In large animal veterinary hospitals, disinfectants have been shown to reduce environmental bacteria (12, 13); however, there is no literature regarding the efficacy of cleaning and disinfection protocols in small animal veterinary clinics and animal shelters.

Animal shelters constitute prime locations that enable the amplification of exposure to infectious agents (14). Animal shelters house animals from different provenances which in turn occupy several locations within shelter facilities before they are rehomed. Thus, it is important to determine whether animal movements within these facilities are associated with widespread dissemination events of pathogens (14). Current guidelines recommend that strict animal movement control in shelters needs to be implemented to avoid the spread of potential nosocomial bacterial pathogens and infectious diseases (15). To date, no studies have investigated the relationship between environmental bacteria and animal movements in animal health care facilities.

This study aimed to (a) uncover the relationship between environmental bacterial contamination and animal movements within the shelter as a potential factor for influencing environmental bacterial levels and (b) investigate the effect of pre- and post-cleaning/disinfection practices in an animal shelter on the presence of potential nosocomial bacterial pathogens, the total bacterial, coliform, and *E*. *coli* levels.

## Materials and Methods

### Ethical Statement

The study was reviewed and approved by the Production and Companion Animal ethics committee of the School of Veterinary Science at the University of Queensland (The University of Queensland Animal Ethics SVS/487/15/KIBBLE).

### Study Setting and Environmental Sampling Design

**Figure 1:**
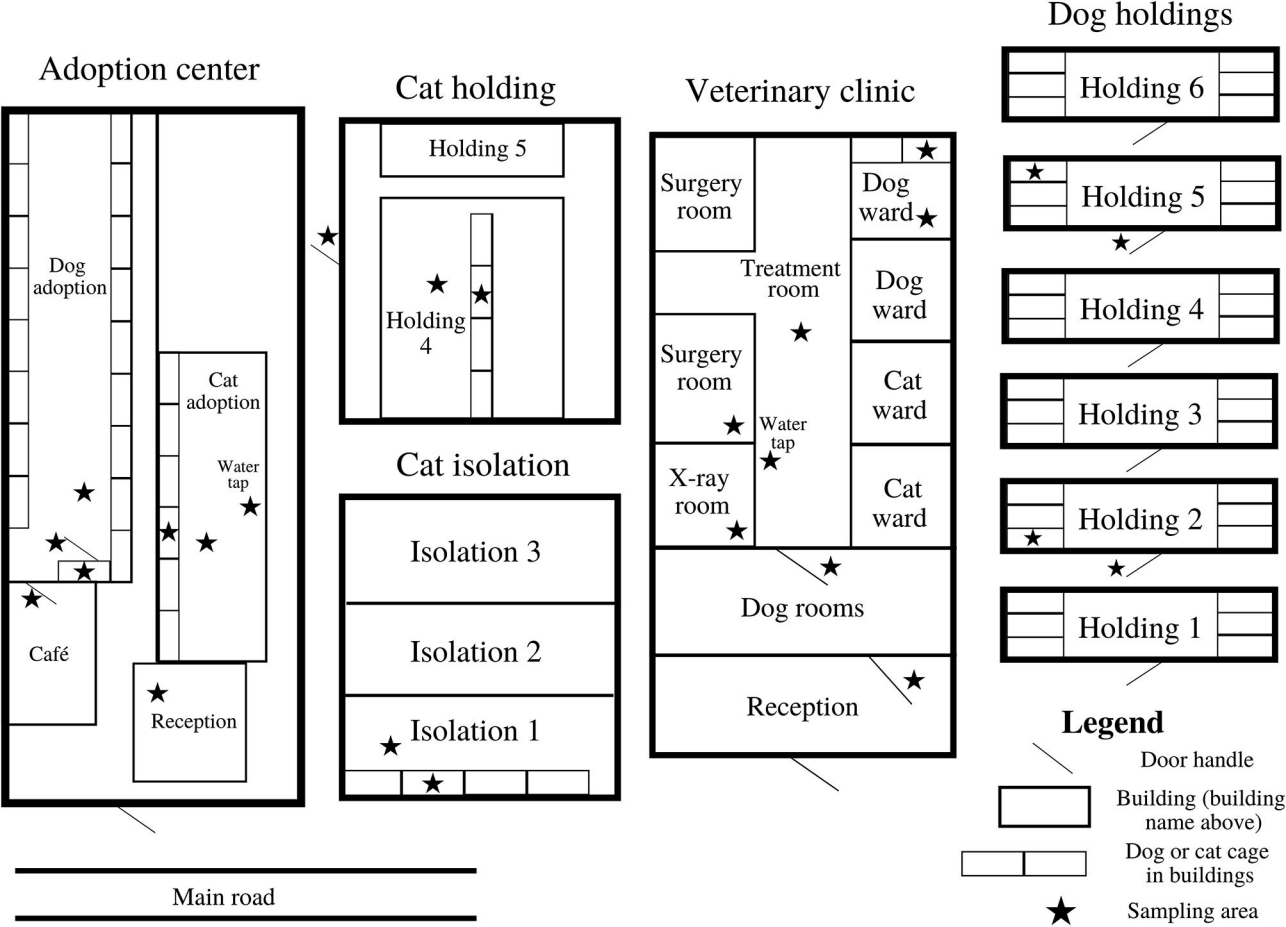
Basic map of the animal shelter showing the sampling areas that were collected from the seven target locations.

The study was performed in the largest animal shelter in Brisbane, Australia, which holds a small animal veterinary clinic, an adoption center and dog and cat holdings (kennels). Environmental sampling occurred from the 30th of April 2018 to the 16th of May 2018. To obtain a representative sampling frame, a total of seven locations with high animal movements within the shelter were targeted for environmental sampling including the small animal veterinary clinic, dog holding two and five, cat isolation one, cat holding four, and the dog and cat adoption centers (Figure 1). Within each of these seven target locations, samples were collected from different sampling areas before and after routine cleaning/disinfection was conducted. In brief, the target sampling areas in the animal holdings and adoption center were the dog cage floors and cat cages, door handles and the floor and drain area for the cat locations. Samples taken in the small animal veterinary clinic included: the dog ward cage, floor areas, plastic door between the treatment room and dog ward, the reception door handle, X-ray table, air-conditioner unit in the surgery room and water tap in the treatment room. The reception door handle had two pre-cleaning only samples per week due to there being no cleaning routine. The air-conditioner unit was only sampled once per week due to the absence of additional cleaning in-between the scheduled quarterly professional disinfection. For the number of samples taken pre- and post-infection control over the 3 weeks of sampling per location, refer to Tables 1, 2.

**Table 1:** Number of samples taken over the 3 weeks of sampling (30th of April 2018 to the 16th of May 2018) per location per sampling area at the animal shelter for the pre- and post-cleaning only samples.

	**Number of samples taken over the 3 weeks**	
**Location and sampling areas**	**Pre-cleaning only**	**Post-cleaning only**
**DOG HOLDING TWO**		
Dog cage one floor	3	3
Door handle to dog cage one	3	3
Dog cage 37 floor^a^	1	1
**DOG HOLDING FIVE**		
Dog cage seven floor	3	3
Door handle to dog cage seven	3	3
Dog holding five main door handle	3	3
**CAT HOLDING FOUR**		
Cat cage 12	3	3
Floor area	3	3
Cat cage 13^a^	1	1
**CAT ISOLATION ONE**		
Floor and drain area near cage	3	3
**DOG ADOPTION CENTER**		
Dog cage three floor	3	3
Door handle to dog cage three	3	3
Walkway floor area	3	3
Café door handle	3	3
Reception desk^b^	3	0
**CAT ADOPTION CENTER**		
Cat cage seven	3	3
Water tap	3	3
Floor and drain area near cage	3	3
**Total number of samples (*****N*** **=** **97)**	**50**	**47**

*Cleaning was performed using only Earth Choice detergent (<10% Coco-Glucoside, <1% Sodium Coco-Sulfate)*.

a *Environmental samples were only taken if there was no animal in the cage at the time of sampling as the cage had previously been cleaned and disinfected. Therefore, dog cage 37 floor and cat cage 13 were only sampled in week three*.

b *The dog adoption center reception desk had only pre-cleaning samples taken as there was no cleaning routine*.

**Table 2:** Number of samples taken over the 3 weeks of sampling (30th of April 2018 to the 16th of May 2018) per location per sampling area at the animal shelter for the pre- and post-disinfection samples.

	**Number of samples taken over the 3 weeks**	
**Location and sampling areas**	**Pre-disinfection**	**Post-disinfection**
**DOG HOLDING TWO**		
Dog cage one floor	1	1
**DOG HOLDING FIVE**		
Dog cage seven floor^a^	0	1
**CAT ISOLATION ONE**		
Cat cage seven	3	3
**CAT ADOPTION CENTER**		
Cat cage seven	2	2
**SMALL ANIMAL VETERINARY CLINIC**		
Dog ward cage	3	3
Dog ward floor area	3	3
Plastic door between treatment room and dog ward	3	3
Treatment room floor area	3	3
X-ray table	3	3
Air-conditioner unit^b^	3	0
Reception door handle^c^	6	0
Water tap in treatment room	3	3
**Total number of samples (*****N*** **=** **58)**	**33**	**25**

*Disinfection was performed after cleaning using Clinikill Concentrate (7.7 g/L Benzalkonium Chloride) and Virkon S (21.41% Potassium peroxymonosulfate, 1.5% Sodium chloride)*.

a *The dog holding five, dog cage seven floor only had a post-disinfection sample as the floor was disinfected prior to sampling*.

b *In the veterinary clinic there were no post-disinfection samples for the air-conditioner unit as it was professionally disinfected prior to sampling. ^c^The reception door handle had no cleaning routine in the veterinary clinic; thus, all samples were classified as pre-disinfection*.

For the animal holdings and adoption center, as cleaning commenced at 7:30 a.m., pre-cleaning and post-cleaning only samples using Earth Choice detergent were taken from 7:30 a.m. to 9:00 a.m. Monday to Wednesday. Due to the distance between the shelter and the laboratory and that disinfection commenced after 3:00 p.m. in the veterinary clinic, samples could not be processed directly after sampling. Post-disinfection samples for the veterinary clinic were taken in the morning, the following day, before animals entered the clinic, 16–18 h after disinfection. No animal movements at the sampled areas occurred between disinfection and sampling in the veterinary clinic. Pre-disinfection samples were taken at midday, which allowed for daily veterinary procedures to occur.

A total of 155 environmental samples were taken during the study. Fifty samples were pre-cleaning only and 47 were post-cleaning only. Thirty-three pre-disinfection and 25 post-disinfection samples were also taken. Three locations had cleaning and disinfectant samples taken over the 3 weeks on different days. Each environmental sample was taken wearing new sterile gloves and wiping the electrostatic wipes (Pascoes Pty Ltd., Fairfield East, Sydney, Australia) over a target surface ~1 m^2^, horizontally and vertically, back and forth for both sides of the wipe for larger sized surfaces such as the floors and cages. For the smaller sized surfaces such as the door handles and water taps, the wipes were wiped over the entire stainless-steel surface. Wipes were then stored in labeled ziplock bags, transported to the laboratory in a cooler box and processed on the same day as collection.

### Cleaning and Disinfection Protocols

Animal shelter staff and volunteers clean the dog and cat holdings and adoption center each morning. The outdoor dog holdings were cleaned while dogs were walked; all items were removed from the cage, for instance, bedding and feces, then Earth Choice detergent (<10% Coco-Glucoside, <1% Sodium Coco-Sulfate; Natures Organics, Australia) was put onto the floor. The floor was pressure hosed, excess water was removed from the cage floor and allowed to dry before the dog was returned to its cage. The indoor dog adoption center had a similar cleaning protocol; however, floors were mopped to ensure all debris was removed and then mopped again using a different mop. For the cat cages in the cat isolation, holding and adoption center, feces, and debris were removed from the litter trays and cages, and Earth Choice detergent was used to clean the cage with a damp paper towel. The cat isolation cages were disinfected daily after cleaning using a 1:50 dilution of Virkon® S for 10 min (21.41% Potassium peroxymonosulfate, 1.5% Sodium chloride; Lanxess, Cologne, Germany). Staff and volunteers wore the same gloves when cleaning multiple cages within the same cat isolation location. All dog and cat cages were disinfected after cleaning using the same dilution and contact time of Virkon® S as above, upon exit of each animal, after a disease incident or weekly, whichever of these events happened first.

The small animal veterinary clinics cleaning and disinfection protocol included removing the animal, bedding, feces, and debris from the cages, washing the cages using a 1:10 dilution of Clinikill® Concentrate for a contact time of 5 min (7.7 g/L Benzalkonium Chloride) which is marketed as a disinfectant, germicidal detergent (Pharmchem, Eagle Farm, Queensland, Australia) and then wiping off any remaining liquid. The cages were air-dried and sprayed with Virkon® S (same concentration and contact time as above). Clinikill® Concentrate was also only used for disinfecting the bench surfaces (including the X-ray table) and the floors. There was no cleaning routine for the reception door handle in the veterinary clinic which was sampled twice per week (six pre-cleaning only samples in total). The air-conditioner unit in the surgery room was sampled by wiping an electrostatic wipe over the air-conditioner blades once per week. The air-conditioner unit was professionally disinfected quarterly, and the last disinfection process had occurred prior to starting the study and did not reoccur during the sampling period, thus, all samples were classified as pre-disinfection. The process of cleaning using only Earth Choice detergent will be referred to as “cleaning only” and the cleaning followed by disinfection using Clinikill® Concentrate only or in conjunction with Virkon® S will be referred to as “disinfection” in this study. Cleaning always occurred prior to disinfection.

### Bacterial Isolation

#### Environmental Bacterial Levels—Total Bacterial, Coliform and *E. Coli* Counts

The wipes were aseptically placed into separate 100 mL of phosphate buffered saline (PBS) (original PBS solution) and were shaken vigorously for 15 s. A 10-fold serial dilution (from 10^−1^ to 10^−2^) was completed using the original PBS solution. To calculate the total viable bacterial count, 100 μL of the original PBS solution and each serial dilution was pipetted in duplicate onto half plates onto separate Plate Count Agar (PCA) (Thermo Fisher Scientific) and incubated aerobically for 24 h at 37°C. The total bacterial count was calculated by counting the individual colonies and averaging the result of the two halves of the PCA plate. To enumerate coliforms and *E*. *coli*, 1 mL of the original PBS solution and the 10^−1^ serial dilution were pipetted onto separate 3M^TM^ Petrifilm^TM^ *E*. *coli*/Coliform Count Plates, processed and identified as per the manufacturer's instructions (16). Colony counts between 0 and 250 were multiplied by the dilution factor to estimate the original solutions colony forming units (CFU/mL). Negative controls for the wipes were completed at the beginning of each new packet. A disinfectant neutralizer (17) was not used in this study as 77.6% (45/58) of the total disinfectant samples were taken from the veterinary clinic.

#### Environmental Bacterial Isolation

After completing the serial dilutions, the original PBS solution was incubated for 24 h at 37°C. To isolate environmental *P*. *aeruginosa*, 10 μL of the post-incubated original PBS solution was used to inoculate the Cetrimide Selective Agar (Thermo Fisher Scientific). After incubation of the Cetrimide Selective Agar for 48 h at 42°C, an oxidase test was performed on any suspect colonies. One milliliter of the post-incubated original PBS solution was pipetted into 9 mL Tryptone Soya broth containing 50 μg/mL of ampicillin (TSB/AMP) for ampicillin resistant *Enterobacteriaceae* isolation and 8 mL Mueller Hinton broth containing 6.5% NaCl (MH/NaCl) for *Staphylococcus* spp. isolation. All broths were incubated at 37°C for 24 h. The TSB/AMP broth was used to inoculate MacConkey agar plates containing 50 μg/mL of ampicillin (MCA/AMP) which were incubated at 37°C for 24 h, as ampicillin resistance is commonly expressed in MDR *Enterobacteriaceae* (18). The MH/NaCl broths were used to inoculate MRSA 2 Brilliance^TM^ agar and were incubated at 37°C for 48 h. After incubation of all selective media, colony morphology was observed, and suspect *P*. *aeruginosa, Enterobacteriaceae* and methicillin resistant *Staphylococcus* spp. (MRS) colonies were sub-cultured onto separate Sheep Blood Agar (SBA) plates (Thermo Fisher Scientific) and incubated for 24 h at 37°C. The isolates were stored in 1 mL of brain heart infusion (BHI) with 20% glycerol in −80°C. Refer to Supplementary Methods for the extended methods.

### Identification of Bacterial Isolates

Prior to identification, isolates were cultured onto SBA and all bacterial isolates were identified using freshly grown overnight colonies using a matrix-assisted laser desorption ionization—time of flight mass spectrometry (MALDI-TOF MS) (Bruker Corporation, Bremen, Germany). Timperio et al. (19) direct colony transfer and DNA extraction methods were used. Although, all suspect MRS spp. isolates were pre-treated with 1 μL of 70% formic acid before adding 1 μL of α-cyano-4-hydroxy-cinnamic acid MALDI matrix. For the DNA extraction method, completed for the isolates with low-confidence and no organism identification, 25 μL of 70% formic acid and 25 μL of 100% acetonitrile were added. Standard Bruker interpretative criteria were used to interpret the results. Briefly, if the bacterial identification scores were 2.00 to 3.00, the results were accepted with high-confidence at the species level (20). If the identification scores were 1.70 to 1.99, the results were classified as low-confidence and were accepted at the genus level only (20). The low-confidence and no organism identification (<1.69) samples were repeated using the DNA extraction method.

### Antimicrobial Susceptibility Testing

Disc diffusion antimicrobial susceptibility testing was performed for the identified isolates according to the Clinical and Laboratory Standards Institute guidelines (21, 22). *P*. *aeruginosa* and ampicillin resistant *Enterobacteriaceae* isolates were tested against 13 antimicrobial agents and MRSP isolates were tested against 14 antimicrobial agents. The quality control strains were *P*. *aeruginosa* ATCC® 27853, *E*. *coli* ATCC® 25922 and *S*. *aureus* ATCC® 25923 (Supplementary Methods).

### Detection of the *mecA* Gene in Methicillin Resistant *Staphylococcus* spp. Using PCR

DNA was extracted from all suspect MRS spp. isolates using a modified version of Garcha et al. (23) Chelex DNA extraction methods. This included firstly pelleting the bacteria in sterilized water, using 6% Chelex matrix (Bio-Rad, Gladesville, New South Wales, Australia) and incubating samples at 100°C for 8 min. The samples were centrifuged at 8,117 × g for 10 min and stored at −80°C.

The PCR reaction mixture consisted of 2 μL DNA, 200 μL Amplitaq Gold 360 Master Mix (Thermo Fisher Scientific) and 20 μM of each primer. The primers for the 310-bp *mecA*-specific products were previously described by Geha et al. (24) (Supplementary Table 4) and PCR amplification was conducted: denaturation at 95°C for 10 min, followed by 10 cycles (94, 65–55, and 72°C each for 1 min), then 25 cycles (94, 53, and 72°C each for 1 min), and a final extension at 72°C for 5 min. *S*. *aureus* ATCC® 25923 (negative control) and *S*. *aureus* ATCC® 43300 (positive control) were used as the quality control strains.

The PCR products (10 μL) were visualized using the GelDoc System (Bio-Rad Laboratories) in 1.5% agarose gel (Bio-Rad), containing 1% sodium borate buffer (Bio-Rad), and stained with SYBR safe (Invitrogen Australia Pty Limited, Sydney, NSW, Australia). For this study, all *Staphylococcus pseudintermedius* isolates that were resistant to oxacillin and contained the *mecA* gene are referred to as MRSP and all other isolates are referred to as *S. pseudintermedius*.

### Animal Movements

All adult dogs and cats are housed individually with separate compartments in the cat and dog cages for the toileting and sleeping areas. Each animals' length of stay and the locations they are moved throughout the shelter is unique. Although, the general dog movements are as follows: incoming dogs are taken to the short-term dog holdings (holding two to four in Figure 1) then moved to the veterinary clinic for an initial clinical assessment. A behavioral assessment is conducted in a separate room in the adoption center and the dogs are moved to the long-term dog holdings (holdings 5–6 in Figure 1). Dogs with no behavioral or medical concerns are transferred to the dog adoption center until they are adopted. The short-term and long-term dog holdings were located in an undercover shelter exposed to the elements and the dog adoption center was located indoors.

Feline patients are initially taken to the veterinary clinic for a clinical examination. If there are no signs of infection during the veterinary assessment, cats are moved to the cat holdings and later transferred to the cat adoption center. If cats have an illness, such as upper respiratory tract signs, then they are transferred to cat isolation. Once they are treated and are no longer showing signs of infection, they may be moved to the cat holdings or to the cat adoption center. The cat holdings, isolation and adoption center were all located indoors. Cats are transported throughout the shelter in a carrier and dogs are walked on a leash when possible. Dogs and cats can also be transferred to different holdings or be relocated within the same location (Figure 1).

### Social Network Analysis of Animal Movements Within the Shelter

Animal movement data from all of the sampled locations during the sampling period were extracted from the shelter database management system and stored in a MS Excel spreadsheet. The MS Excel spreadsheet included the following data: unique animal identification number, name and breed, first shelter location along with the cage number and the date that the animal was moved into that location, the new shelter location including the cage number and the date the animal was moved into the next location, and the date that the animals left the shelter. The individual animal movements were collated to determine the total number of animal movements between shelter locations using the animal identification numbers, shelter locations and dates. All areas that animals had moved to and the environmental sampling target locations were included using an uni-directed network of animal movements. Animals transferred to another cage in the same location was kept as separate data and not used for the social network analysis. An MS Excel spreadsheet with all of the animal movement data was imported into the UCINET software package (Analytic Technologies, Lexington, Kentucky, USA). Two measures of connectivity were estimated for each shelter location including the degree centrality and *K*-core. The degree centrality measure is an individual measure of connectivity which measures the number of unique links from a given location to other locations; for example, a location with an estimated degree centrality of five indicates that the site is connected to five other sites (25). A *K*-core is a group measure of connectivity in which each node is connected to the same number of other nodes in the group (25).

### Statistical Analyses

The relationship between the bacterial levels (CFU/mL) and cleaning and disinfection practices were quantified using a negative binomial regression model for the total bacterial and coliform levels. The colony counts were not transformed into log counts due to the over-dispersion of the bacterial levels illustrated in Supplementary Figures 1, 2. As such, a negative binomial model was chosen to account for the over-dispersion and its adequacy was evaluated using the nbvargr command in Stata version 13.1.

A random effect of sample location was added to account for multiple samples taken in each of the seven locations. Within the seven locations, 13 sampled surfaces were selected for the negative binomial regression models which included: both dog holding cage floors, cat isolation one and cat holding four cage and floor areas, dog adoption cage floor and walkway floor, cat adoption cage and floor and drain area, and the clinics' dog ward floor, treatment room floor, and X-ray table. The dog holding two cage floor and the cat adoption cage both had cleaning only and disinfection samples in this analysis. To detect changes in the bacterial levels pre- and post-cleaning/disinfection, locations with higher total bacterial and coliform levels were selected for analysis. Statistical analyses were not completed for the *E*. *coli* levels due to low or no bacterial levels. *K*-core was not included in this dataset for analysis as all seven sampled target locations were part of the same sub-graph with a *K*-core value of four. All statistical analyses were performed using Stata 13.1 (Stata Corporation, College Station, TX, USA).

To identify potential factors which may influence the levels of bacteria in the environment, the relationship between the total bacterial levels (outcome of Model 1) and the coliform levels (outcome of Model 2) and the variables of interest were quantified using separate negative binomial regression models. The variables of interest included: the degree centrality values, the total number of animal movements throughout the shelter and the total number of animals moving to a different cage within the same location. The negative binomial model was sensitive to the presence of outliers in the bacterial level data. In some instances (5.9%; 5/85 samples included), the post-cleaning total bacterial counts were >1,000 CFU/mL, likely due to contamination as an animal was present in the cage when the sample was taken. The post-cleaning total bacterial counts >1,000 CFU/mL were removed from the dataset for analysis due to this reason. Confounding variables were assessed by observing their impact on the coefficient of other variables by >25%. The final model was selected based on the smallest estimate of the Akaike's Information Criterion (AIC).

## Results

### Presence of Bacteria Pre- and Post-cleaning and/or Disinfection in the Animal Shelter Environment

Both dog holdings, cat isolation one floor and drain area, cat holding four, and dog and cat adoption centers samples using Earth Choice detergent were included in the cleaning only results (Table 1). The disinfection results were taken from the small animal veterinary clinic, both dog holding cage floors, cat isolation one cage and the cat adoption cage (Table 2).

For the overall isolation of bacteria pre- and post-infection control, refer to Table 3. Bacteria were isolated from both dog holding cage floors and the main door handle to dog holding two, cat adoption cage and the floor and drain area, cat holding four cage, and floor area and the dog adoption cage floor, walkway floor, café door handle, and the reception desk (Supplementary Table 1). Eleven percent (5/47) of the post-cleaning only samples were removed due to animal contamination. In the veterinary clinic, bacteria were isolated from all areas, except the reception door handle and treatment room water tap (Supplementary Tables 2, 3). None of the control wipes had any visible bacterial growth on the selective media nor Petrifilm^TM^.

**Table 3:** Total number of isolates identified using MALDI-TOF which were isolated from different environmental sampling areas pre- and post-cleaning only and disinfection.

**Isolates**	**Cleaning only samples (%)**		**Disinfection samples (%)**	
	**Pre-cleaning only (*N* = 50)**	**Post-cleaning only (*N* = 47)**	**Pre-disinfection (*N* = 33)**	**Post-disinfection (*N* = 25)**
*Pseudomonas aeruginosa* (*n* = 36)	10 (20)	11 (23.4)	7 (21.2)	8 (32)
*Escherichia coli* (*n* = 12)	3 (6)	5 (10.6)	3 (9.1)	1 (4)
*Enterobacter* spp. (*n* = 20)	5 (10)	7 (14.9)	4 (12.1)	4 (16)
*Klebsiella* spp. (*n* = 26)	12 (24)	8 (17)	3 (9.1)	3 (12)
Methicillin resistant *Staphylococcus pseudintermedius* (*n* = 3)	2 (4)	0 (0)	1 (3)	0 (0)
*Staphylococcus pseudintermedius* (*n* = 1)	0 (0)	0 (0)	1 (3)	0 (0)

*Methicillin resistant Staphylococcus pseudintermedius isolates were resistant to oxacillin and contained the mecA gene. The Staphylococcus pseudintermedius isolate was resistant to oxacillin without the presence of the mecA gene*.

*N, total number of samples taken pre- and post-cleaning only or disinfection; n, total number of isolates identified using the MALDI-TOF*.

### Antimicrobial Susceptibility Results

Five of the 36 *P*. *aeruginosa* isolates could not be recovered after MALDI-TOF identification. The same antibiogram was shared between 77% (24/31) of the *P*. *aeruginosa* isolated from different locations in the shelter (Table 4). Eighty-seven percent of the *P*. *aeruginosa* isolates were not susceptible to ticarcillin (27/31) and 97% to ticarcillin/clavulanic acid (30/31). Only 10% (3/31) of the *P*. *aeruginosa* isolates were MDR as defined by Magiorakos et al. (26).

**Table 4:** *Pseudomonas aeruginosa* and ampicillin resistant *Enterobacteriaceae* isolates which were cultured from different environmental sampling areas within the animal shelter displaying resistance to the antimicrobial drugs.

**Isolates**		**Antimicrobial drugs**																
		**TE**	**IMP**	**C**	**CAZ**	**FEP**	**AK**	**GEN**	**SXT**	**TIC**	**AMC**	**AMP**	**CEF**	**ENR**	**CIP**	**TZP**	**TIM**	**PRL**
*Pseudomonas aeruginosa* (*N* = 31)	*n* (%)	31^a^ (100)	0 (0)	31^a^ (100)	1 (3)	0 (0)	0 (0)	2 (6)	31^a^ (100)	27 (87)	NT	NT	NT	NT	0 (0)	1 (3)	30 (97)	1 (3)
*Escherichia coli* (*N* = 12)	*n* (%)	6 (50)	3 (25)	4 (33)	2 (16)	0 (0)	2 (16)	2 (16)	8 (66)	12 (100)	10 (83)	12 (100)	12 (100)	1 (8)	NT	NT	NT	NT
*Enterobacter* spp. (*N* = 19)	*n* (%)	9 (47)	6 (32)	8 (42)	8 (42)	2 (11)	0 (0)	3 (16)	11 (58)	17 (89)	19^a^ (100)	19^a^ (100)	19^a^ (100)	8 (42)	NT	NT	NT	NT
*Klebsiella* spp. (*N* = 25)	*n* (%)	4 (16)	0 (0)	6 (24)	3 (12)	2 (8)	1 (4)	3 (12)	3 (12)	25^a^ (100)	5 (20)	25^a^ (100)	7 (28)	3 (12)	NT	NT	NT	NT

*AK, amikacin (30 μg); AMC, amoxicillin/clavulanic acid (20/10 μg); AMP, ampicillin (10 μg); FEP, cefepime (30 μg); CAZ, ceftazidime (30 μg); CEF, cephalothin (30 μg); C, chloramphenicol (30 μg); CIP, ciprofloxacin (5 μg); ENR, enrofloxacin (5 μg); GEN, gentamicin (10 μg); IMP, imipenem (10 μg); PRL, piperacillin (100 μg); TZP, piperacillin/tazobactam (100/10 μg); TE, tetracycline (30 μg); TIC, ticarcillin (75 μg); TIM, ticarcillin/clavulanic acid (75/10 μg); SXT, trimethoprim/sulfamethoxazole (1.25/23.75 μg)*.

a *isolates with intrinsic resistance, N, total number of isolates; n, number of isolates displaying resistance (including intermediate and resistant isolates); NT, not tested*.

*Five P. aeruginosa, one Enterobacter spp., and one Klebsiella spp. isolates were unrecoverable after MALDI-TOF identification*.

Two of the 58 ampicillin resistant *Enterobacteriaceae* isolates could not be recovered after MALDI-TOF identification. All *Enterobacteriaceae* isolates were resistant to ampicillin (Table 4). Only 10% (2/19) of the *Enterobacter* spp. and none of the *E*. *coli* isolates shared the same antibiograms but were resistant to several antibiotics. At least 47% of the *Enterobacter* spp. and *E*. *coli* isolates were resistant to the following antibiotics: cephalothin (100% for both bacteria), amoxicillin/clavulanic acid (100 and 83%, respectively), ticarcillin (89 and 100%, respectively), trimethoprim/sulfamethoxazole (58 and 66%, respectively), tetracycline (47 and 50%, respectively). Sixty percent (15/25) of the *Klebsiella* spp. shared the same antibiogram and were only resistant to ampicillin and ticarcillin. Resistance to other antibiotic classes ranged between 0 and 42%. MDR was detected in 58% (11/19) of *Enterobacter* spp., 91% (11/12) of *E*. *coli* and 20% (5/25) of *Klebsiella* spp. isolates (26).

The MALDI-TOF identified four environmental *S. pseudintermedius* isolates. The *mecA* gene was only identified in three of the four environmental *S. pseudintermedius* and these were classed as MRSP. The MRSP isolates were isolated from the cat isolation one floor and drain area, dog holding five cage floors and the cage in the treatment room. The *S*. *pseudintermedius* isolate lacking the *mecA* gene was isolated from the dog holding two cage floor. Two MRSP and the *S*. *pseudintermedius* isolates shared the same antibiogram which were isolated from the dog holding two and five cage floors and the cat isolation floor and drain area. The MRSP isolates were all MDR (100%; 3/3) (26) (Table 5).

**Table 5:** Methicillin resistant *S. pseudintermedius* (*N* = 3) and *S. pseudintermedius* (*N* = 1) isolates which were cultured from different environmental sampling areas within the animal shelter displaying resistance to the antimicrobial drugs.

**Isolates**		**Antimicrobial drugs**													
		**AK**	**AMC**	**C**	**DA**	**ENR**	**E**	**GEN**	**IMP**	**MUP**	**OX**	**P**	**TE**	**SXT**	**VA**
Methicillin resistant *S. pseudintermedius* (*N* = 3)	*n* (%)	0 (0)	3 (100)	3 (100)	3 (100)	3 (100)	3 (100)	0 (0)	0 (0)	0 (0)	3 (100)	3 (100)	3 (100)	3 (100)	0 (0)
*S. pseudintermedius* (*N* = 1)	*n* (%)	0 (0)	1 (100)	1 (100)	1 (100)	1 (100)	1 (100)	0 (0)	0 (0)	0 (0)	1 (100)	1 (100)	1 (100)	1 (100)	0 (0)

*AK, amikacin (30 μg); AMC, amoxicillin/clavulanic acid (20/10 μg); C, chloramphenicol (30 μg); DA, clindamycin (2 μg); ENR, enrofloxacin (5 μg); E, erythromycin (15 μg); GEN, gentamicin (10 μg); IMP, imipenem (10 μg); MUP, mupirocin (200 μg); OX, oxacillin (1 μg); P, penicillin (10 units); TE, tetracycline (30 μg); SXT, trimethoprim/sulfamethoxazole (1.25/23.75 μg); VA, vancomycin (30 μg)*.

*N, total number of isolates; n, number of isolates displaying resistance (including intermediate and resistant isolates)*.

### Social Network Analysis of Animal Movements Within the Animal Shelter

**Figure 2:**
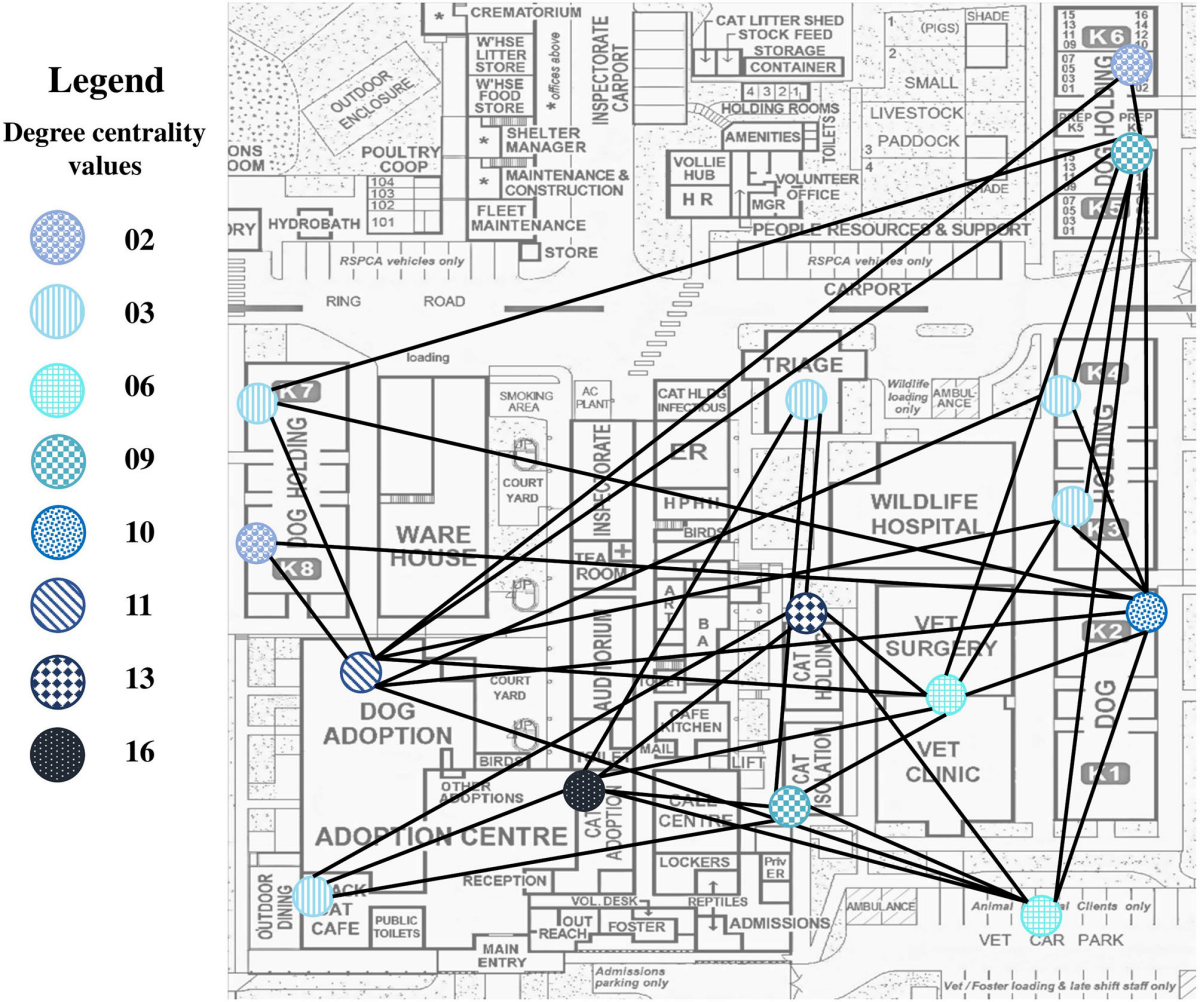
Partial map of the animal shelter with the social network using the degree centrality values. This map was provided by the animal shelter and the social networking diagram created in UCINET was adapted for this map. Each colored circle correlates to a degree centrality value for the different locations within the shelter. Some of the nodes were omitted as they were either grouped together, for instance, cat isolation included cat isolation 1–3 or they were not present on this map. Not to scale.

The seven sampled locations shared the same *K*-core value of four, indicating that each of the seven locations had ties to at least four other locations. The degree centrality measurement extracted from the social network diagram was adapted for the partial map of the animal shelter (Figure 2). Each node (colored circle) in Figure 2 corresponds to a degree centrality measure and indicates that the animal shelter was highly connected by dog and cat movements. For the sampled locations, the cat adoption center had the highest degree centrality value of 16 in this network. The cat holding four, dog adoption center, and dog holding two had high degree centrality values of 13, 11, and 10, respectively. Cat isolation one and dog holding five had a degree centrality value of 9 and the small animal veterinary clinic and the animals that had left the shelter both had a degree centrality value of 6 (Figure 2).

### Relationship Between Environmental Bacterial Levels, Infection Control Procedures, and Animal Movements in the Facility

When combining the cleaning only and disinfection sample results, the total bacterial counts were reduced post-cleaning only and post-disinfection (Coef. = −1.44, 95% CI = −2.06, −0.81, and *P* ≤ 0.001) (Table 6). Without accounting for timing, there was less total bacterial (Coef. = −1.72, 95% CI = −3.09, −0.34, and *P* = 0.015) and coliform contamination (Coef. = −1.44, 95% CI = −2.08, −0.80, and *P* ≤ 0.001) with disinfection when compared to cleaning only. There was a reduction in the total bacterial count in week 2 compared with the results from week 1 which was statistically significant (Table 6). When factoring in the degree centrality measure, the sampled areas that were less connected by animal movements were more likely to be at-risk of total bacterial (Coef. = −0.32, 95% CI = −0.49, −0.15, and *P* ≤ 0.001; Table 6) and coliform contamination (Coef. = −0.14, 95% CI = −0.22, −0.06, and *P* = 0.001; Table 7). The sampled areas with less animals changing cages within the same animal holding had an increased risk of coliform contamination (Coef. = −0.05, 95% CI = −0.08, −0.02, and *P* = 0.001; Table 7).

**Table 6:** Multivariable negative binomial regression for the total bacterial count in the environment of 13 different sampling areas within the seven target locations in the animal shelter and the effect on cleaning and/or disinfection.

**CFU/mL**	**Coeff. (95% CI)**	***p*-value**
**TIMING**		
Pre-infection control	Reference	
Post-infection control	−1.44 (−2.06, −0.81)	≤0.001
**INFECTION CONTROL**		
Cleaning only	Reference	
Cleaning and disinfection	−1.72 (−3.09, −0.34)	0.015
**WEEKS**		
Week 1	Reference	
Week 2	−0.98 (−1.87, −0.09)	0.03
Week 3	−0.48 (−1.93, 0.96)	0.514
**ANIMAL MOVEMENTS**		
Degree centrality	−0.32 (−0.49, −0.15)	≤0.001
Number of animal movements within the same location	0.02 (−0.02, 0.06)	0.262
Intercept	10.17 (7.56, 12.79)	≤0.001
/Inalpha	1.00 (0.54, 1.45)	NT

*This table was adapted from the results in Stata version 13.1. For timing, infection control includes pre- and post-cleaning and pre- and post- disinfection samples. For weeks, the baseline data was the pre- and post-cleaning only and disinfection colony forming units from week 1. The interaction between timing and infection control was not conducted due to the minimal disinfection samples. CFU, colony forming units per milliliters; Week 2, cleaning results of week 2 compared with week 1; Week 3, cleaning results of week 3 compared with week 1; NT, not tested*.

**Table 7:** Multivariable negative binomial regression for the coliform count in the environment of 13 different sampling areas within the seven target locations in the animal shelter and the effect on cleaning and/or disinfection.

**CFU/mL**	**Coeff. (95% CI)**	***p*-value**
**TIMING**		
Pre-infection control	Reference	
Post-infection control	0.02 (−0.65, 0.69)	0.951
**INFECTION CONTROL**		
Cleaning only	Reference	
Cleaning and disinfection	−1.44 (−2.08, −0.80)	≤0.001
**WEEKS**		
Week 1	Reference	
Week 2	−0.17 (−1.20, 0.86)	0.748
Week 3	−0.15 (−1.32, 1.01)	0.794
**ANIMAL MOVEMENTS**		
Degree centrality	−0.14 (−0.22, −0.06)	0.001
Number of animal movements within the same location	−0.05 (−0.08, −0.02)	0.001
Intercept	5.07 (3.65, 6.50)	≤0.001
/Inalpha	1.02 (0.55, 1.50)	NT

*This table is adapted from the results in Stata version 13.1. For timing, infection control includes pre- and post-cleaning and pre- and post- disinfection samples. For weeks, the baseline data was the pre- and post-cleaning only and disinfection colony forming units from week 1. The interaction between timing and infection control was not conducted due to the minimal disinfection samples. CFU, colony forming units per milliliters; Week 2, cleaning results of week 2 compared with week 1; Week 3, cleaning results of week 3 compared with week 1; NT, not tested*.

## Discussion

To the best of our knowledge, this is the first study to investigate the effects of cleaning only and disinfection practices at reducing bacterial levels in the environment of an animal shelter that also houses a veterinary clinic. Our study revealed the presence of MRSP, *E*. *coli, Enterobacter* spp., *Klebsiella* spp. and *P*. *aeruginosa* in the environment of the animal shelter and veterinary clinic with a significant proportion of *Enterobacter* and *E. coli* being MDR. Our results also indicate that while contamination levels are generally sensitive to cleaning and disinfection procedures, a lack of animal movement within the premises correlates to an increased level of contamination likely due to lesser disinfection practices. Taken together our results highlight the importance of cleaning and disinfection guidelines in a mixed facility such as the one investigated here and the potential for clinic movement data to be used as an indicator of potential environmental bacterial contamination.

Our results indicated that the total bacterial count in the environment of the sampled locations were generally reduced post-cleaning only and post-disinfection. Overall, there was less total bacterial contamination with the disinfection samples when compared to the cleaning only samples. Even though, the effect of timing and weeks of sampling was not statistically significant, it is important to note there was less coliform contamination with the disinfection samples as well. The total bacterial counts >1,000 CFU/mL post-cleaning only can partly be explained by the fact that mops were not cleaned after every use and animals had returned to their cages before a sample could be taken which likely re-contaminated the area. Additionally, *E*. *coli, Enterobacter* spp., *Klebsiella* spp., and *P*. *aeruginosa* were still isolated from the environment of the animal holdings and adoption center's post-cleaning only and from the veterinary clinic post-disinfection. Detergent has minimal effect on reducing environmental bacteria as it is commonly used to remove surface debris (27), and with contact from animals, staff and volunteer's shoes and cleaning equipment, bacteria may have continually been re-introduced into the environment. Bacteria present in the veterinary clinic post-disinfection was possibly due to insufficient cleaning before disinfection, human contamination via shoes and resistances to disinfectants. Other reasons could include incorrect disinfection procedures, for instance, incorrect mixing and contact time as Virkon® S and Clinikill® Concentrate both recommend a 1:100 dilution rate and a contact time of 10 min to be effective at eliminating environmental bacteria (28, 29). In addition, disinfecting surfaces frequently touched by humans such as the café door handle and low animal traffic areas, and changing the water in the buckets and cleaning the mops after every cage should be conducted to ensure that there is a decrease in the bacterial load. Overall, these result show that cleaning followed by disinfection is essential to reduce the total bacterial and coliform counts and the presence of potential nosocomial bacterial pathogens.

Most *P*. *aeruginosa* isolates were resistant to ticarcillin/clavulanate (97%; 30/31), a drug commonly used as a last resort to treat *P. aeruginosa* infections. All *Enterobacteriaceae* isolates cultured in this study were resistant to ampicillin and to a broad variety of antibiotics, including extended spectrum cephalosporin, ceftazidime (23%; 13/56). The *S*. *pseudintermedius* isolate that was *mecA* negative was phenotypically resistant to oxacillin. Thus, further molecular and phenotypic testing or genotyping could be conducted to determine if there was a loss of the mobile genetic element (30), resistance to methicillin via the *mecC* gene (31) or if the isolate was phenotypically resistant to methicillin via β-lactamase hyper-production reported in MRSA, respectively (32). The same bacteria, with the same antibiogram (*P*. *aeruginosa*: 77%; 24/31 isolates, *Enterobacteriaceae*: 30%; 17/56 isolates, and MRSP and *S*. *pseudintermedius*: 75%; 3/4 isolates) were cultured from different locations throughout the shelter. To determine genetic relatedness, BOX-PCR and enterobacterial repetitive intergenic consensus (ERIC)-PCR could be conducted for the *P*. *aeruginosa* and *Enterobacteriaceae* isolates (33, 34). However, these methods have low intra- and inter-laboratory reproducibility, are unable to discriminate between highly related *P*. *aeruginosa* isolates and have a long analysis time, making them suboptimal methods for large investigations (33, 35, 36). Thus, future studies may be able to conduct whole genome sequencing and phylogenetic studies to further identify clonal spread of organisms and the transfer of mobile genetic elements between bacteria in a shelter environment. MRSP (100%; 3/3), *P*. *aeruginosa* (10%; 3/31) and *Enterobacteriaceae* (48%; 27/56) isolates were classified as MDR and were often resistant to critically important antibiotics. MDR bacteria pose an infection risk to animals but also a risk to veterinary personnel and owners. Thus, knowing the presence of such resistant bacteria in the environment justifies the use and compliance of solid infection control protocols.

Our results demonstrate that the effect of post-cleaning only and post-disinfection was confounded by the level of animal movements within the shelter. As such, the sampled areas that were less connected by animal movements (the veterinary clinic, dog holding five, and cat isolation one) were more likely to have higher levels of total bacterial and coliform contamination. This result can partly be explained by the fact that the samples in the veterinary clinic were taken 16–18 h after disinfection which could have allowed enough time for some re-contamination from minimal human movements to occur. Further, dog holding five and cat isolation one was classified as the “long-term” animal holdings in this study. Animals stayed in those areas for a longer time period with less animals moving to and from those locations. Dog holding five and the cat isolation one floor was likely to only be cleaned daily and disinfected once per week. The sampled areas with less animals changing cages within the same location had a higher risk of coliform contamination. This is possibly due to the fact that upon exit of each animal, whether to a different location entirely or changing cages within the same location, the areas were cleaned and disinfected. Thus, it is important to ensure that cleaning and disinfection practices are conducted appropriately even if there are fewer animal movements within the same location as these may constitute reservoirs of contamination.

The findings of our study need to be interpreted in light of a few limitations. Firstly, this study was designed as a population-based investigation and thereby was limited by the shelters cleaning and disinfection schedules and practices which influenced how and when samples were taken. Additionally, as sampling occurred over 3 consecutive weeks and the same locations were sampled, the animal shelter personnel behaviors may have changed as they became more conscious of how they were cleaning and disinfecting. This could have introduced biases in our data. For example, it was not always possible to take all samples before the animals returned to their cages as all locations had the same morning cleaning schedule. However, to minimize this, samples from the same target locations, for instance, dog holding two and five were taken on the same day each week to increase the likelihood that a post-cleaning only or a post-disinfection sample could be taken. Additionally, any confounders were evaluated in the statistical model. Secondly, the time it took for the bacterial counts to return to its pre-cleaning levels was not investigated as this study focused on the initial findings of the effects of pre- and post-infection control only. Future studies could investigate the changes in bacterial levels at different time intervals to determine if the cleaning and disinfection frequency needs to be increased. Thirdly, we did not screen for the *mecC* gene in the *Staphylococcus* spp. isolates and no whole genome sequencing was conducted on any of the isolates which hampered our ability to investigate the presence of bacterial clones in different locations and sampling timings. Lastly, only a select number of locations were sampled, thus, future studies should delve deeper using all locations with animal movements and account for staff behavior with respect to cleaning and disinfection. This will allow for the investigation into the effect of animal movements on environmental bacterial contamination over a longer period and a larger sampling size.

## Conclusion

Overall, this study demonstrates that cleaning followed by disinfection practices reduced the total bacterial and coliform levels in the shelter environment. As animal connectivity within the shelter influences environmental bacterial levels, it highlights the need for shelter staff and volunteers to target areas with fewer animal movements when cleaning and disinfecting to reduce the risk of surfaces acting as environmental reservoirs of transmission and infection.

## Data Availability Statement

The raw data supporting the conclusions of this article will be made available by the authors, without undue reservation.

## Ethics Statement

The animal study was reviewed and approved by the Production and Companion Animal ethics committee of the School of Veterinary Science at the University of Queensland (The University of Queensland Animal Ethics SVS/487/15/KIBBLE). Written informed consent was obtained from the owners for the participation of their animals in this study.

## Author Contributions

EM, JG, and RSM conceived and designed the experiments. SH and HR performed the experiments. SH, HR, and XZ analyzed the data. All authors contributed to the manuscript and read and approved the submitted version.

## Conflict of Interest

The authors declare that the research was conducted in the absence of any commercial or financial relationships that could be construed as a potential conflict of interest.

## Supplementary Material

Supplementary Table 4 Primer sequences incorporating mutations of amino acids 74 and 129 in mink and raccoon dog SLAM genes.
