# Supplementary material for: Environmental Recovery of Nosocomial Bacteria in a Companion Animal Shelter Before and After Infection Control Procedures
Source: Front Vet Sci. 2021 Jan 20;7:608901. doi: 10.3389/fvets.2020.608901 (PMC7854535; doi:10.3389/fvets.2020.608901)
Supplement: Supplementary Table 4 — Primer sequences incorporating mutations of amino acids 74 and 129 in mink and raccoon dog SLAM genes. [file Data_Sheet_3.docx]

Supplementary Material

# Supplementary methods

## Bacterial isolation

### Environmental bacterial levels – total bacterial, coliform and *E*. *coli* counts

The wipes were placed into separate 100 mL of phosphate buffered saline (PBS) to make the original solution and were shaken vigorously for 15 seconds. A 10-fold serial dilution (from 10^-1^ to 10^-2^) was completed using the original PBS solution. To calculate the total viable bacterial count, 100 µL of the original solution and each serial dilution was pipetted in duplicate onto half plates onto separate Plate Count Agar (PCA) (Thermo Fisher Scientific) and incubated aerobically for 24 hours at 37°C. The total bacterial count was calculated by counting the individual colonies and averaging the result of the two halves of the PCA plate. For the coliform and *E*. *coli* counts, 1 mL of the original PBS and the 1/10 serial dilution were pipetted onto separate 3M^TM^ Petrifilm^TM^ *E*. *coli*/Coliform Count Plates (Thermo Fisher Scientific), incubated for 48 hours at 37°C and the colonies were identified using the manufacturers interpretation guide.

For the PCA plates and Petrifilm colony counts, the dilutions with colonies between 0 and 250 were counted and multiplied by the dilution factor to estimate the colony forming units (CFU) in the original solution (reported as CFU/mL).

### Environmental bacterial isolation

After completing the serial dilutions, the original PBS solution was incubated for 24 hours at 37°C. For the isolation of environmental *P*. *aeruginosa*, Cetrimide Selective Agar (Thermo Fisher Scientific) was streaked using the previously incubated original PBS solution and incubated for 48 hours at 42°C, then identified using colony morphology and an oxidase test.

Environmental ampicillin resistant *Enterobacteriaceae* was isolated using Tryptone Soya broth containing 50 µg/mL of ampicillin which was inoculated with 1 mL of the original PBS solution and incubated for 24 hours at 37˚C. The broth was streaked onto the MacConkey agar plates containing 50 µg/mL of ampicillin, incubated for 24 hours at 37˚C and each phenotypically different lactose fermenting colony type indicated as a pink colony was recorded.

For the isolation of environmental MRSP and MRSA, Mueller Hinton broth containing 6.5% NaCl (MH/NaCl broth) was inoculated with 1 mL of the original PBS, incubated for 24 hours at 37°C, then streaked onto MRSA 2 Brillance^TM^ agar which were incubated for 48 hours at 37°C. All dark blue denim (suspect MRSA) and light blue denim (suspect MRSP) colonies were recorded.

All suspect *P*. *aeruginosa*, ampicillin resistant *Enterobacteriaceae*, MRSP and MRSA colonies were sub-cultured onto Sheep Blood Agar (SBA) plates (Thermo Fisher Scientific) and incubated for 24 hours at 37˚C. The bacterial colonies were stored in separate microcentrifuge tubes containing 1 mL of brain heart infusion (BHI) with 20% glycerol (Thermo Fisher Scientific) in -80˚C.

## Antimicrobial susceptibility testing

The following methods were used for the interpretation of the breakpoints for the methicillin resistant *Staphylococcus* spp. isolates. For mupirocin, no zone of inhibition was interpreted as resistant and any sized zone of inhibition was interpreted as susceptible. Imipenem was interpreted using the performance standards for antimicrobial susceptibility testing on Oxoid’s website (Oxoid 2013). For vancomycin, no zone of inhibition was classed as resistant and minimum inhibitory concentrations tests should be performed to confirm susceptibility.

The article by MacKenzie et al. (2004) was used to interpret zone diameters for chloramphenicol and tetracycline as it provided information on the breakpoints for the *Pseudomonas aeruginosa* isolates.

## Detection of the *mecA* gene in methicillin resistant *Staphylococcus* spp.

All suspect methicillin resistant *Staphylococcus* spp. isolates were sub-cultured onto SBA from the brain heart infusion broth and incubated for 24 hours at 37°C. A bacterial colony was suspended in a microfuge tube containing 1 mL of sterilised water using a sterile 10 µL loop. The sample was centrifuged for one minute at 8117 x g and the supernatant was discarded. The bacteria were resuspended in 6% Chelex matrix (Bio-Rad, Gladesville, New South Wales, Australia) and incubated at 56°C for 20 minutes. The samples were vortexed, incubated at 100°C for eight minutes and then cooled in ice for five minutes. The samples were centrifuged at 8117 x g for a further ten minutes and stored at –80°C for later use.

A polymerase chain reaction (PCR) was performed to identify the presence or absence of the *mecA* gene. The molecular methodology was based on the previously used techniques in the study by Geha et al. (1994). The control organisms used in this study included: *S*. *aureus* ATCC® 25923 (negative control) and *S*. *aureus* ATCC® 43300 (positive control).

Before performing the PCR, the DNA samples were retrieved from the -80°C freezer, allowed to thaw, and then centrifuged at 11337 x g for two minutes. The concentrations of the DNA products were identified using a NanoDrop^TM^ 1000 Spectrophotometer (Thermo Fisher Scientific) prior to being added to the PCR reaction mixture and the concentrations of each isolate varied from 21-220 µg/µL. The reaction mixture for the PCR was formulated and consisted of 2 µL DNA, 200µL Amplitaq Gold 360 Master Mix (Thermo Fisher Scientific) and 20 µM of each primer in a total volume of 250 µL. The primers used to identify the *mecA* gene are identified in Supplementary Table S4. The reaction was amplified by MyCycler^TM^ Thermal Cycler System (Bio-rad). The amplification was carried out using a Touchdown PCR program: initial denaturation at 95°C for 10 min was followed by 10 cycles of amplification (denaturation at 94°C for 1 min, 65-55°C for 1 min and 72°C for 1 min). The next amplification was 25 cycles (94°C for 1 min, 53°C for 1 min and 72°C for 1 min), ending with the final extension at 72°C for 5 min. The samples were stored at -20°C until required.

The PCR products were thawed and 10 µL of the products were run on a 1.5% agarose gel (Bio-Rad), containing 1% sodium borate buffer (Bio-Rad) at 60 volts for 90 minutes. The gel was stained using SYBR safe (Invitrogen Australia Pty Limited, Sydney, NSW, Australia) and visualised using the GelDoc System (Bio-Rad Laboratories). Further testing would need to be conducted on the isolates with no *mecA* band to determine if the mobile genetic element was lost after DNA extraction or if there were other resistance mechanisms encoding resistance to methicillin.

**Supplementary Table S4.** PCR primers used to identify the *mecA* gene

| Primers for *mecA* PCR | Primer name | Sequence (5’-3’) | Size of PCR product |
| --- | --- | --- | --- |
| *mecA* gene forward | GMEC 1 | GTAGAAATGACTGAACGTCCGATAA | 310 bp |
| *mecA* gene reverse | GMEC 2 | CCAATTCCACATTGTTTCGGTCTAA |  |

This table was adapted from the information in the study by Geha et al. (1994).

**References**

Geha, D. J., J. R. Uhl, C. A. Gustaferro, and D. H. Persing. 1994. "Multiplex PCR for identification of methicillin-resistant staphylococci in the clinical laboratory." *J. Clin. Microbiol.* 32 (7):1768-1772. doi: 10.1128/JCM.32.7.1768-1772.1994.

MacKenzie, F. M., S. V. Smith, K. E. Milne, K. Griffiths, J. Legge, and I. M. Gould. 2004. "Antibiograms of resistant Gram-negative bacteria from Scottish CF patients." *J. Cyst. Fibros.* 3 (3):151-157. doi: 10.1016/j.jcf.2004.03.009.

Oxoid. 2013. "CLSI and FDA table update." accessed 26th September. <http://www.oxoid.com/pdf/uk/2013-CLSIFDA-table-update.pdf>.
