# Supplementary material for: Environmental Recovery of Nosocomial Bacteria in a Companion Animal Shelter Before and After Infection Control Procedures
Source: Front Vet Sci. 2021 Jan 20;7:608901. doi: 10.3389/fvets.2020.608901 (PMC7854535; doi:10.3389/fvets.2020.608901)
Supplement: Supplementary file 2 [file Data_Sheet_1.docx]

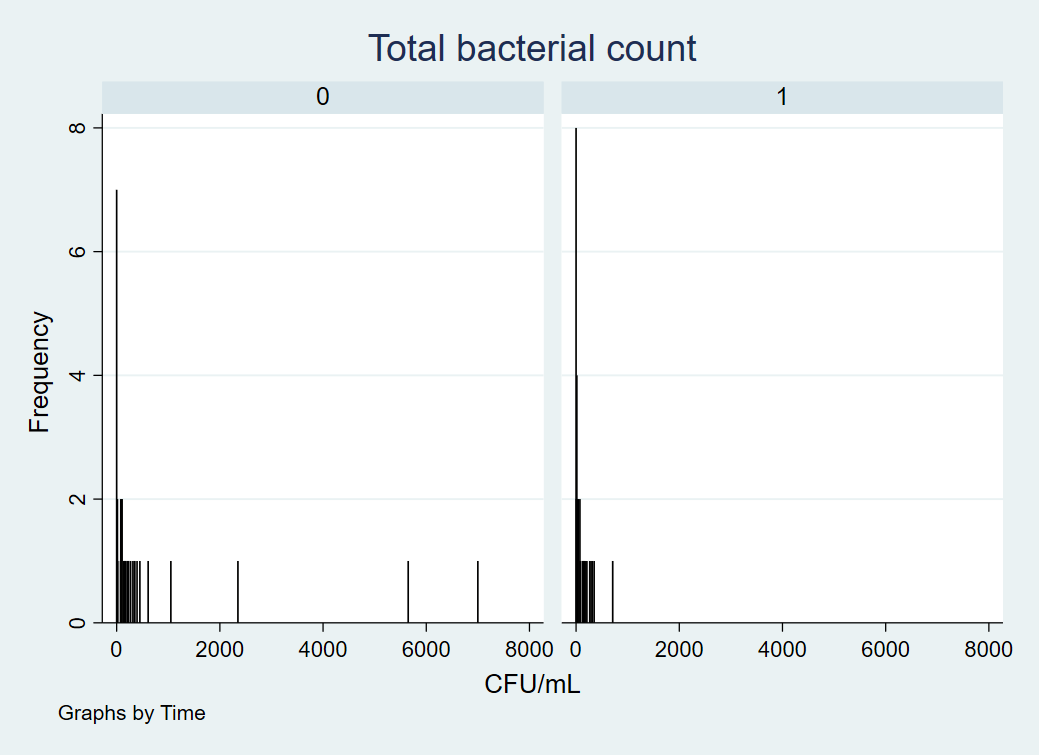


Supplementary Figure S1. Histogram displaying the overdispersion of the total bacterial count pre- and post-infection control by the colony forming units per milliliters (CFU/mL).
0 = pre-infection control, 1 = post-infection control. Figure created using Stata version 15.1.


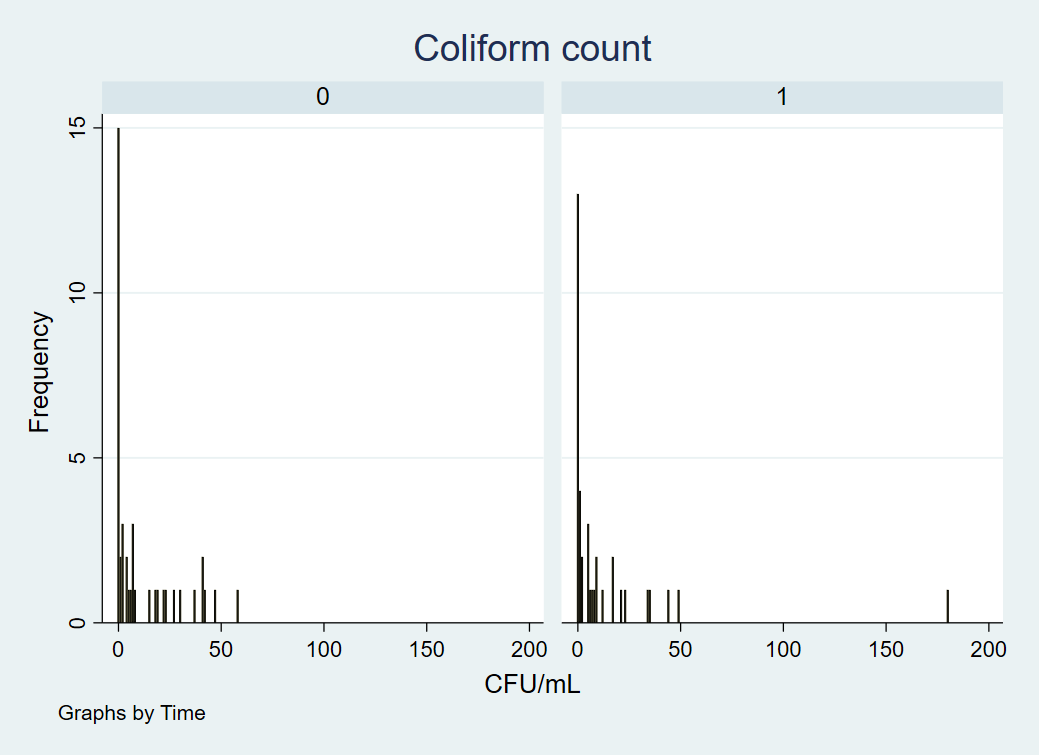


Supplementary Figure S2. Histogram displaying the overdispersion of the coliform count pre- and post-infection control by the colony forming units per milliliters (CFU/mL). 0 = pre-infection control, 1 = post-infection control. Figure created using Stata version 15.1.
