# Supplementary material for: Environmental Recovery of Nosocomial Bacteria in a Companion Animal Shelter Before and After Infection Control Procedures
Source: Front Vet Sci. 2021 Jan 20;7:608901. doi: 10.3389/fvets.2020.608901 (PMC7854535; doi:10.3389/fvets.2020.608901)
Supplement: Supplementary file 3 [file Data_Sheet_2.docx]

**Supplementary Table S1.** Bacteria present pre- and post- cleaning only over the three weeks of environmentally sampling in each location of the dog and cat holdings and the dog and cat adoption center of the animal shelter

| **Environmental site sampled** | **Week 1** | | **Week 2** | | **Week 3** | |
| --- | --- | --- | --- | --- | --- | --- |
|  | **Pre-cleaning** | **Post-cleaning** | **Pre-cleaning** | **Post-cleaning** | **Pre-cleaning** | **Post-cleaning** |
| **Cat holding four cage** | *Enterobacter cloacae* | *Klebsiella pneumoniae,*  *Pseudomonas aeruginosa* | NC | NC | NC | NC |
| **Cat holding four floor** | *Pseudomonas aeruginosa* | *Pseudomonas aeruginosa* | *Klebsiella pneumoniae* | *Pseudomonas aeruginosa* | *Enterobacter cloacae,*  *Pseudomonas aeruginosa* | *Enterobacter cloacae,*  *Pseudomonas aeruginosa* |
| **Cat isolation one drain and floor** | *Enterobacter cloacae* | *Enterobacter cloacae* | *Klebsiella pneumoniae,*  MRSP^[[1]](#footnote-1)^ | *Escherichia coli,*  *Klebsiella pneumoniae* | NC | NC |
| **Cat adoption cage** | *Enterobacter cloacae* | NC | NC | *Enterobacter cloacae* | *Enterobacter asburiae* | AC |
| **Cat adoption drain and floor** | NC | *Enterobacter cloacae,*  *Escherichia coli* | *Pseudomonas aeruginosa* | *Enterobacter asburiae,*  *Pseudomonas aeruginosa* | *Escherichia coli* | NC |

No bacteria were isolated from the tap near the entrance to the room of the cat adoption center. NC = negative culture. AC = animal contamination (sample removed).

**Supplementary Table S1 continued.**

| **Environmental site sampled** | **Week 1** | | **Week 2** | | **Week 3** | |
| --- | --- | --- | --- | --- | --- | --- |
|  | **Pre-cleaning** | **Post-cleaning** | **Pre-cleaning** | **Post-cleaning** | **Pre-cleaning** | **Post-cleaning** |
| **Dog holding two cage floor** | *Klebsiella pneumoniae*,  *Pseudomonas aeruginosa* | AC | *Klebsiella pneumoniae*, *Klebsiella oxytoca* | *Klebsiella pneumoniae*, *Pseudomonas aeruginosa* | *Escherichia vulneris* | *Klebsiella pneumoniae*,  *Pseudomonas aeruginosa* |
| **Dog holding two main door handle** | NC | *Klebsiella oxytoca* | NC | NC | NC | NC |
| **Dog holding five cage floor** | *Pseudomonas aeruginosa* | *Escherichia coli, Klebsiella pneumoniae*,  *Pseudomonas aeruginosa* | *Klebsiella pneumoniae*,  *Pseudomonas aeruginosa*,  MRSP | *E*. *aerogenes*,  *Klebsiella pneumoniae*,  *Pseudomonas aeruginosa* | *Enterobacter cloacae*,  *Pseudomonas aeruginosa* | *Pseudomonas aeruginosa* |
| **Dog adoption cage floor (DA3)** | *Klebsiella pneumoniae* | AC | *Klebsiella pneumoniae*,  *Pseudomonas aeruginosa* | AC | *Escherichia coli*,  *Pseudomonas aeruginosa* | NC |
| **Dog adoption walkway floor near DA3** | NC | *Escherichia coli*, *Klebsiella pneumoniae* | NC | *Escherichia coli* | *Klebsiella oxytoca* | *Enterobacter asburiae*,  *Pseudomonas aeruginosa* |
| **Dog adoption cage floor (DA37)** | NT | NT | NT | NT | *Escherichia coli* | AC |
| **Door handle to the café (adoption center)** | *Klebsiella oxytoca* | NC | *Klebsiella oxytoca* | NC | NC | NC |
| **Adoption center reception desk** | *Klebsiella pneumoniae* | NT | NC | NT | NC | NT |

No bacteria were isolated for the dog holding five main door handle and the dog adoption door handle (DA3). DA37 was only sampled if no dog was present in DA3 at the time of sampling. DA = dog adoption with cage number, NC = negative culture, NT = not tested. AC = animal contamination (sample removed).

**Supplementary Table S2.** Bacteria present pre- and post-disinfection over the three weeks of environmentally sampling in each location in the animal shelters’ small animal veterinary clinic

| **Environmental site sampled** | **Week 1** | | **Week 2** | | **Week 3** | |
| --- | --- | --- | --- | --- | --- | --- |
|  | **Pre-disinfection** | **Post-disinfection** | **Pre-disinfection** | **Post-disinfection** | **Pre-disinfection** | **Post-disinfection** |
| **Dog ward floor** | *Escherichia coli*,  *Klebsiella pneumoniae*,  *Pseudomonas aeruginosa* | NC | NC | *Enterobacter cloacae*, *Pseudomonas aeruginosa* | *Escherichia coli*,  *Klebsiella pneumoniae*,  *Pseudomonas aeruginosa* | *Enterobacter asburiae*,  *Klebsiella pneumoniae*,  *Pseudomonas aeruginosa* |
| **Door between treatment room and dog ward** | NC | NC | NC | *Enterobacter cloacae* | NC | NC |
| **Dog ward cage treatment room** | NC | NC | *Pseudomonas aeruginosa* | NC | *Escherichia coli*,  *Pseudomonas aeruginosa*,  MRSP | NC |
| **Floor in treatment room** | *Klebsiella pneumoniae* | *Klebsiella oxytoca*,  *Pseudomonas aeruginosa* | *Enterobacter cloacae* | NC | *Pseudomonas aeruginosa* | *Klebsiella pneumoniae*,  *Pseudomonas aeruginosa* |
| **X-ray table** | *Escherichia coli* | *Escherichia coli* | NC | *Pseudomonas aeruginosa* | *Klebsiella pneumoniae* | NC |
| **Air-conditioner** | NC | NT | *Enterobacter cloacae*,  *Pseudomonas aeruginosa* | NT | NC | NT |

No bacteria were isolated from the water tap in the treatment room of the veterinary clinic and the door handle to and from reception.
NC = negative culture, NT = not tested.

**Supplementary Table S3.** Bacteria present pre- and post-disinfection in the dog holding two and five cage floor, cat isolation one cage and cat adoption cage

| **Environmental site sampled** | **Week 1** | | **Week 2** | | **Week 3** | |
| --- | --- | --- | --- | --- | --- | --- |
|  | **Pre-disinfection** | **Post-disinfection** | **Pre-disinfection** | **Post-disinfection** | **Pre-disinfection** | **Post-disinfection** |
| **Dog holding two cage floor (Virkon)** | *Pseudomonas aeruginosa*,  *Staphylococcus pseudintermedius^[[2]](#footnote-2)^* | *Pseudomonas aeruginosa*,  *Enterobacter cloacae* | NT | NT | NT | NT |
| **Dog holding five cage floor** | NT | *Klebsiella pneumoniae* | NT | NT | NT | NT |
| **Cat adoption cage (Virkon)** | NC | NC | NC | NC | NT | NT |
| **Cat isolation one cage (Virkon)** | NC | NC | NC | NC | *Enterobacter cloacae* | *Pseudomonas aeruginosa* |

In week one, a pre- and post-cleaning and disinfection sample of the dog holding two cage floor and the cat adoption cage room were taken. NC = negative culture, NT = not tested.

1. MRSP = Methicillin resistant *Staphylococcus pseudintermedius* (all *S*. *pseudintermedius* isolates that were resistant to oxacillin and contained the *mecA* gene). [↑](#footnote-ref-1)
2. *Staphylococcus* *pseudintermedius* = all *S*. *pseudintermedius* that were phenotypically resistant to oxacillin without the presence of the *mecA* gene. [↑](#footnote-ref-2)
